# Supplementary material for: Aberrant androgen action in prostatic progenitor cells induces oncogenesis and tumor development through IGF1 and Wnt axes
Source: Nat Commun. 2022 Jul 28;13:4364. doi: 10.1038/s41467-022-32119-0 (PMC9334353; doi:10.1038/s41467-022-32119-0)
Supplement: Supplementary file 1 — Supplementary Information [file 41467_2022_32119_MOESM1_ESM.pdf]

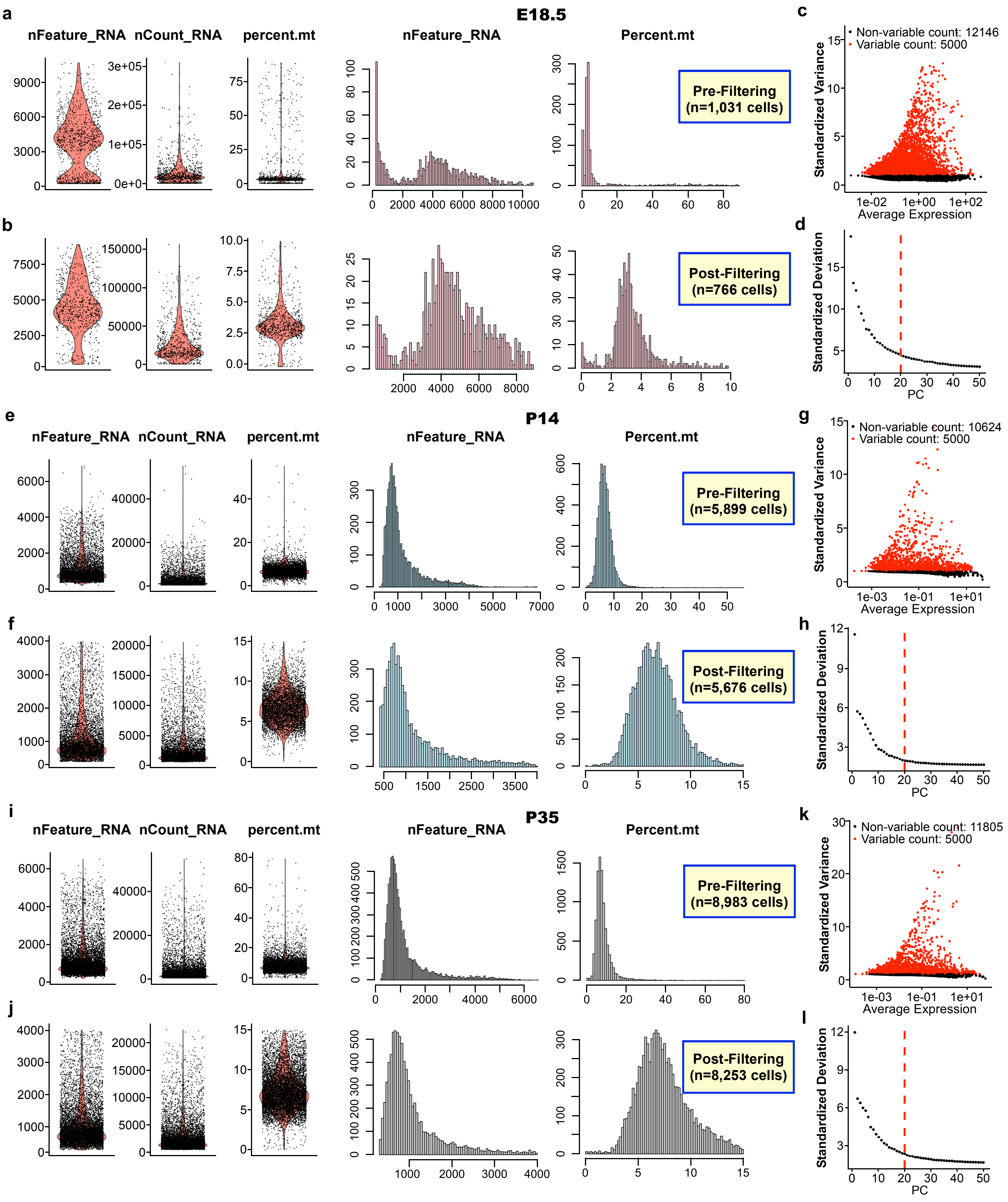

Supplementary Fig. 1 (Related to Fig. 1). Single cell RNA-sequencing quality controls for the E18.5 UGS and P14 or P35 prostate tissues. a Violin and histogram plots corresponding to the number of features, counts, and the percentage of mitochondrial RNA in each cell from E18.5 male UGS before filtering. **b** Violin and histogram plots corresponding to the number of features, counts, and the percentage of mitochondrial RNA in each cell from E18.5 male UGS after filtering for 500 < nFeature < 9,000 and percent.mt < 10. **c** Plot of gene variability and selection of the top 5,000 variable genes from E18.5 male UGS **d** PCA elbow plot identifying a cutoff of 20 dims used for further analyses of E18.5 male UGS. **e** Violin and histogram plots corresponding to the number of features, counts, and the percentage of mitochondrial RNA in each cell from P14 prostate tissues before filtering. **f** Violin and histogram plots corresponding to the number of features, counts, and the percentage of mitochondrial RNA in each cell from P14 prostate tissues after filtering for 400 < nFeature < 4,000 and percent.mt < 15. **g** Plot of gene variability and selection of the top 5,000 variable genes from P14 prostate samples. **h** PCA elbow plot identifying a cutoff of 20 dims used for further analyses of P14 prostate samples. **i** Violin and histogram plots corresponding to the number of features, counts, and the percentage of mitochondrial RNA in each cell from P35 prostate tissues before filtering. **j** Violin and histogram plots corresponding to the number of features, counts, and the percentage of mitochondrial RNA in each cell from P35 prostate tissues after filtering for 300 < nFeature < 4,000 and percent.mt < 15. **k** Plot of gene variability and selection of the top 5,000 variable genes from P35 prostate samples. **l** PCA elbow plot identifying a cutoff of 20 dims used for further analyses of P35 prostate samples.

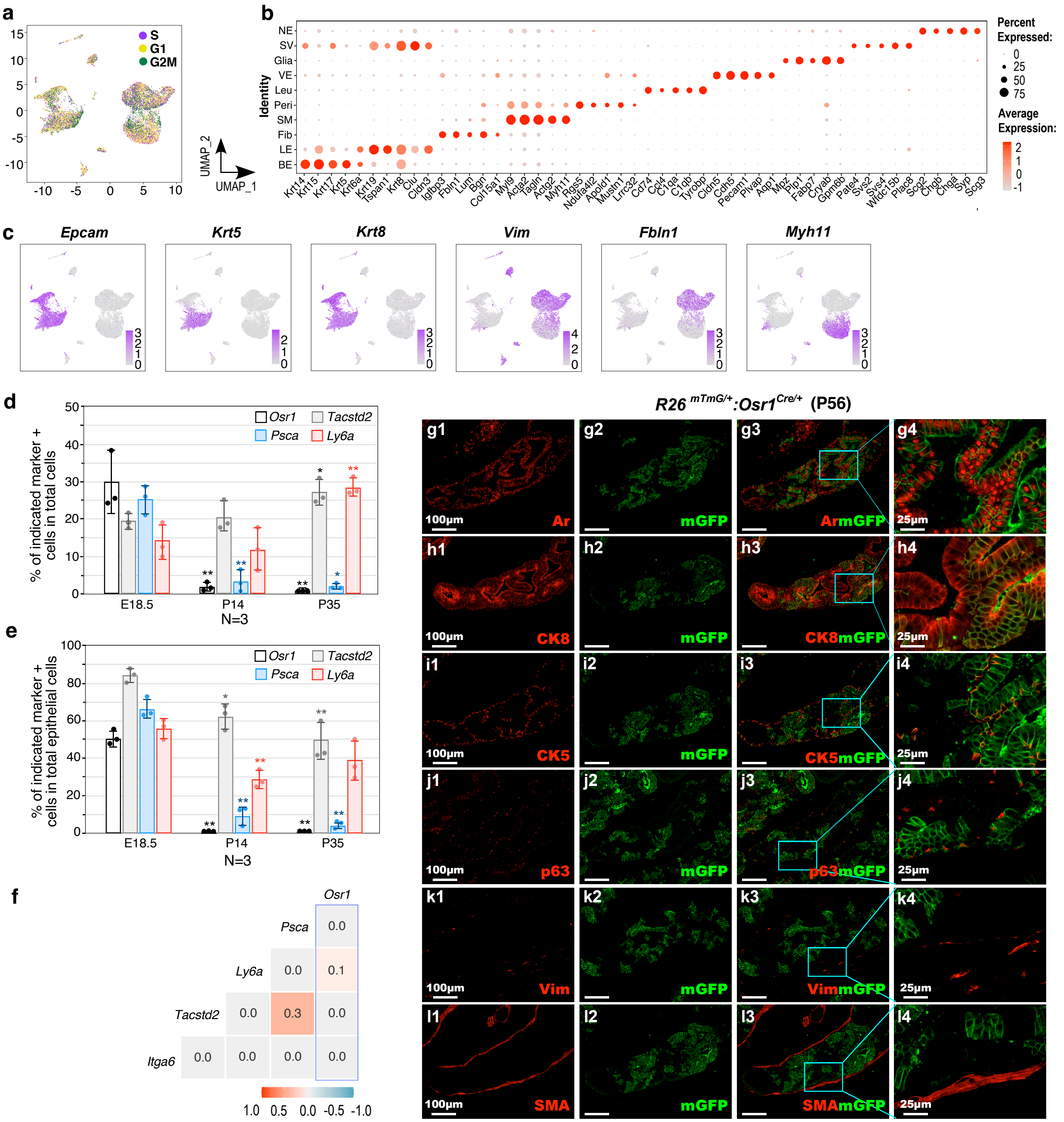

Supplementary Fig. 2 (Related to Fig. 1). Cellular properties of prostatic *Osr1*-lineage cells in embryonic and pubescent development. a UMAP plot displaying the cell cycle phase for each single cell following cell cycle regression. **b** Dot plot displaying five highly specific genes expressed in each cell type from **Fig. 1b**. Size and color of the dot indicates the percentage of cells expressing each gene and the average expression of each gene, respectively. NE, neuroendocrine; SV, seminal vesicle; Glia, glial cells; VE, vascular endothelium; Leu, leukocytes; Peri, pericytes; SM, smooth muscle cells; Fib: fibroblasts; LE: luminal epithelium; BE: basal epithelium. **c** Gene expression UMAP plots of the indicated cell type-specific makers used to determine the identity of each cell cluster from **Fig. 1b**. **d-e** Bar chart showing the percentage of the indicated marker in total cells (**d**) and in epithelial cells (**e**) from E18.5 male UGS, and P14 and P35 prostate tissues in **Fig. 1c**. Data are represented as mean ± SD of three biological replicates. Two-sided *t*-test for E18.5 vs P14 or P35. $*p < 0.05$, $**p < 0.01$. **f** Heatmap of pairwise Spearman correlation between the indicated gene expression in stromal cells from E18.5 male UGS. The number indicates the correlation coefficient. The blue box highlights the correlation between *Osr1* and the indicated progenitor cell markers. **g-l** Representative co-IF staining for the indicated antibodies in adjacent P56 prostate tissue sections from *R26* $^{mTmG/+}$*:Osr1* $^{Cre/+}$ mice. Scale bars, 100 μm (**g1-3, h1-3, i1-3, j1-3, k1-3, l1-3**); 25 μm (**g4, h4, i4, j4, k4, l4**). Nuclei were stained with DAPI. Representative images with consistent results from three independent experiments are shown. Source data and the exact *p*-values are provided in the Source Data file.

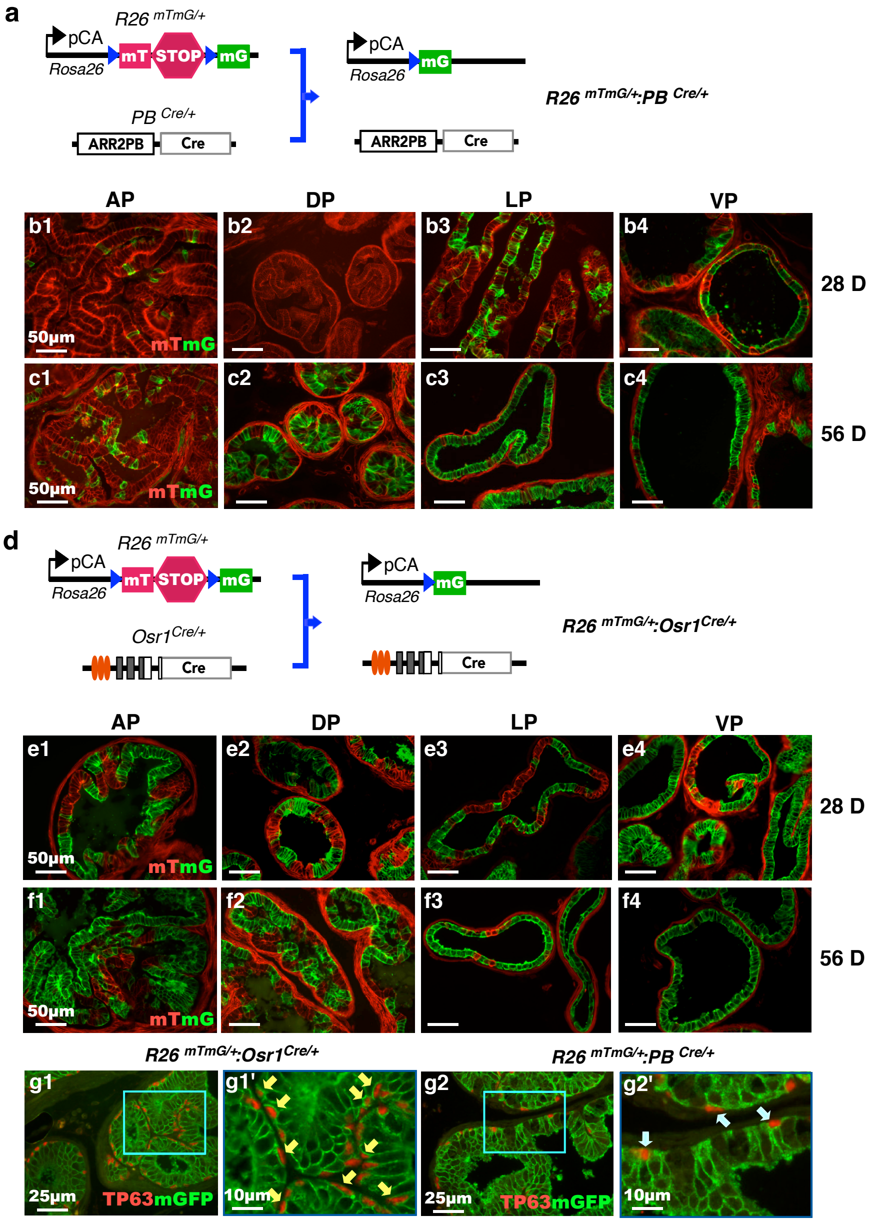

Supplementary Fig. 3 (Related to Fig. 1). Prostatic *Osr1*-lineage cells possess basal epithelial characteristics. a Schematic of $R26^{mTmG/+}$ and $PB^{Cre/+}$ alleles, shown in relation to the mating strategy for this experiment. Constructs are shown for the targeted $R26^{mTmG/+}$ allele undergoing Probasin (*PB*) driven *Cre* mediated recombination in *PB* expressing cells resulting in a change from red to green fluorescence. **b-c** Representative mTmG fluorescence images in P28 and P56 prostate tissue sections from $R26^{mTmG/+}$:$PB^{Cre/+}$ mice. AP: anterior lobe, DP: dorsal lobe, LP, lateral lobe, VP: ventral lobe. Scale bars, 50 µm. **d** Schematic of $R26^{mTmG/+}$ and $Osr1^{Cre/+}$ alleles, shown in relation to the mating strategy for this experiment. D=Days. **e-f** Representative mTmG fluorescence images in P28 and P56 prostate tissue sections from $R26^{mTmG/+}$:$Osr1^{Cre/+}$ mice. Scale bars, 50 µm. **g** Representative images of co-IF for TP63, a basal cell marker, and mGFP in P56 prostate tissue sections from $R26^{mTmG/+}$:$Osr1^{Cre/+}$ or $R26^{mTmG/+}$:$PB^{Cre/+}$ mice. Yellow arrows or blue arrows show double positive cells for TP63 and mGFP or single positive cells that are positive for TP63 and negative for mGFP, respectively. Scale bars, 25 µm (**g1**-**2**); 10 µm (**g1'**-**2'**). Representative images with consistent results from three independent experiments are shown.

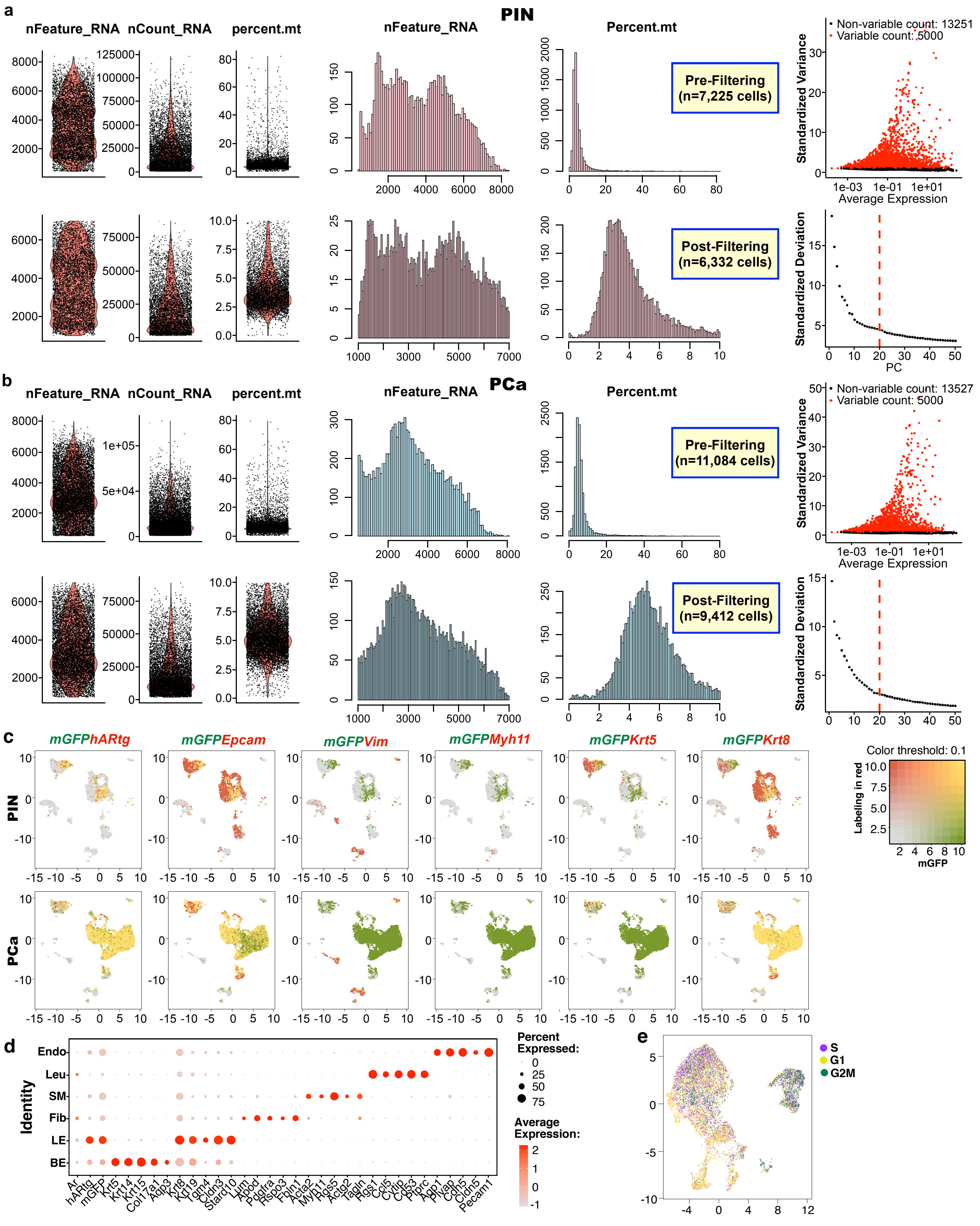

Supplementary Fig. 4 (Related to Fig. 2). Single cell RNA-sequencing quality controls and analyses of the cellular properties from prostatic PIN and tumor tissues induced by *hARtg*. **a** Violin and histogram plots corresponding to the number of features, counts, and the percentage of mitochondrial RNA in each cell from PIN tissues of $R26^{mTmG/hAR}$:$Osr1^{Cre/+}$ mice before filtering. Violin and histogram plots corresponding to the number of features, counts, and the percentage of mitochondrial RNA in each cell after filtering for 1,000 < nFeature < 7,000 and percent.mt < 10. Plot of gene variability and the selection of the top 5,000 variable genes. PCA elbow plot identifying a cutoff of 20 dims used for further analyses. **b** Violin and histogram plots corresponding to the number of features, counts, and the percentage of mitochondrial RNA in each cell from PCa tissues of $R26^{mTmG/hAR}$:$Osr1^{Cre/+}$ mice before filtering. Violin and histogram plots corresponding to the number of features, counts, and the percentage of mitochondrial RNA in each cell after filtering for 1,000 < nFeature < 7,000 and percent.mt < 10. Plot of gene variability and selection of the top 5,000 variable genes. PCA elbow plot identifying a cutoff of 20 dims used for further analyses. **c** Blended expression UMAP plots displaying the expression of mGFP with the indicated genes. PIN (top); PCa (bottom). **d** Dot plot of *Ar*, *hARtg*, *mGFP*, as well as five cluster-specific genes for each cell cluster from PIN and PCa tissues of $R26^{mTmG/hAR}$:$Osr1^{Cre/+}$ mice. **e** UMAP plot displaying the cell cycle phase for each single cell following cell cycle regression for the sub-clustered and re-clustered epithelial cells.

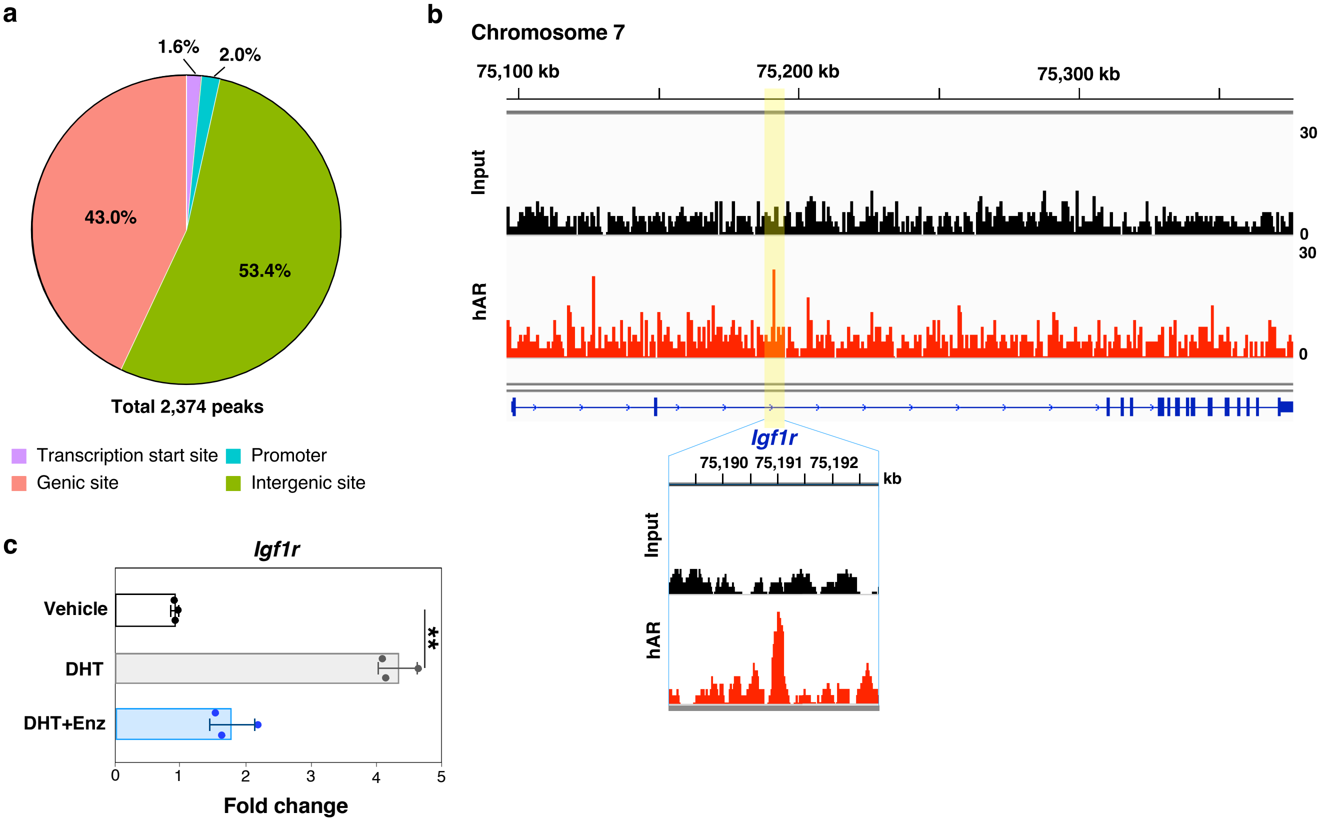

Supplementary Fig. 5 (Related to Fig. 3). IGF1R profile in relation with human AR activation. a Genomic location of hAR binding sites in PIN tissues from *R26*$^{mTmG/hAR}$:*Osr1*$^{Cre/+}$ mice. **b** hAR ChIP-seq enrichment profiles for PIN tissues from *R26*$^{mTmG/hAR}$:*Osr1*$^{Cre/+}$ mice at mouse *Igf1r* locus. **c** RT-qPCR analysis of *Igf1r* expression shown as fold change in organoids derived from PIN tissues from *R26*$^{mTmG/hAR}$:*Osr1*$^{Cre/+}$ mice. Eight days after culture, organoids were treated with vehicle, 1 nM Dihydrotestosterone (DHT), or combination of 1nM DHT and 10 μM enzalutamide (Enz), and were collected for RNA samples at 24 hours after treatment. Data are represented as mean ± SD of three biological replicates. Two-sided *t*-test for DHT vs vehicle, $^{**}p < 0.01$. In **a** and **b**, all data were acquired from two independent experiments with two biological duplicates. Source data and the exact *p*-values are provided in the Source Data file.

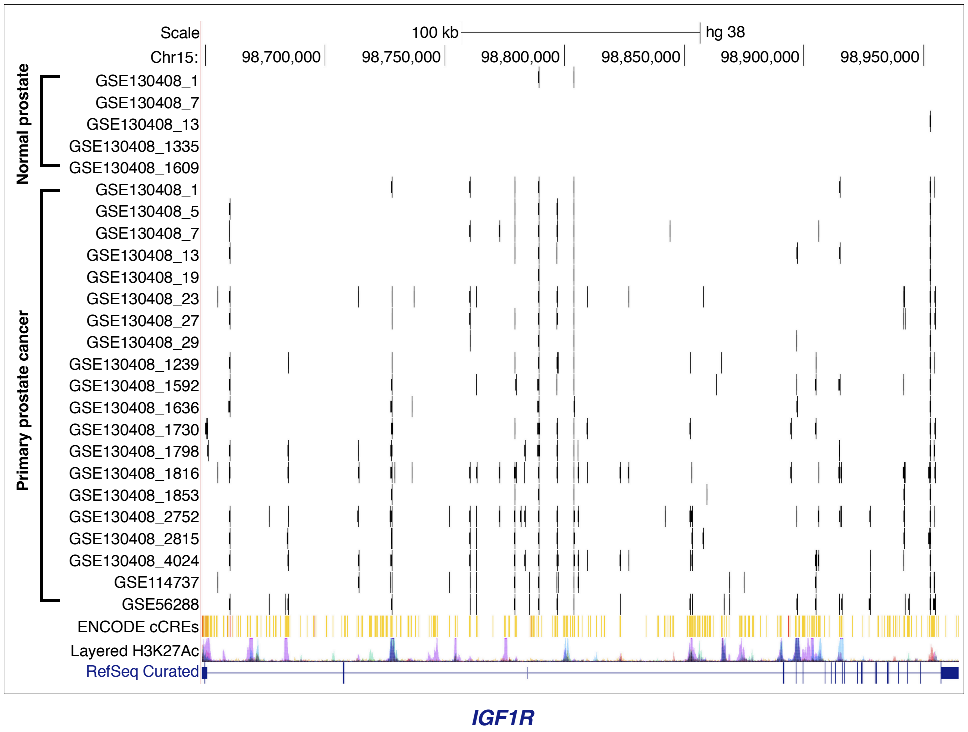

Supplementary Fig. 6 (Related to Fig. 7). Enrichment of AR binding on human *IGF1R* locus in human prostate cancer. a AR ChIP-seq enrichment profiles for human normal prostate or primary prostate cancer tissues on human *IGF1R* locus, visualized with ENCODE and RefSeq tracks on UCSC genome browser.

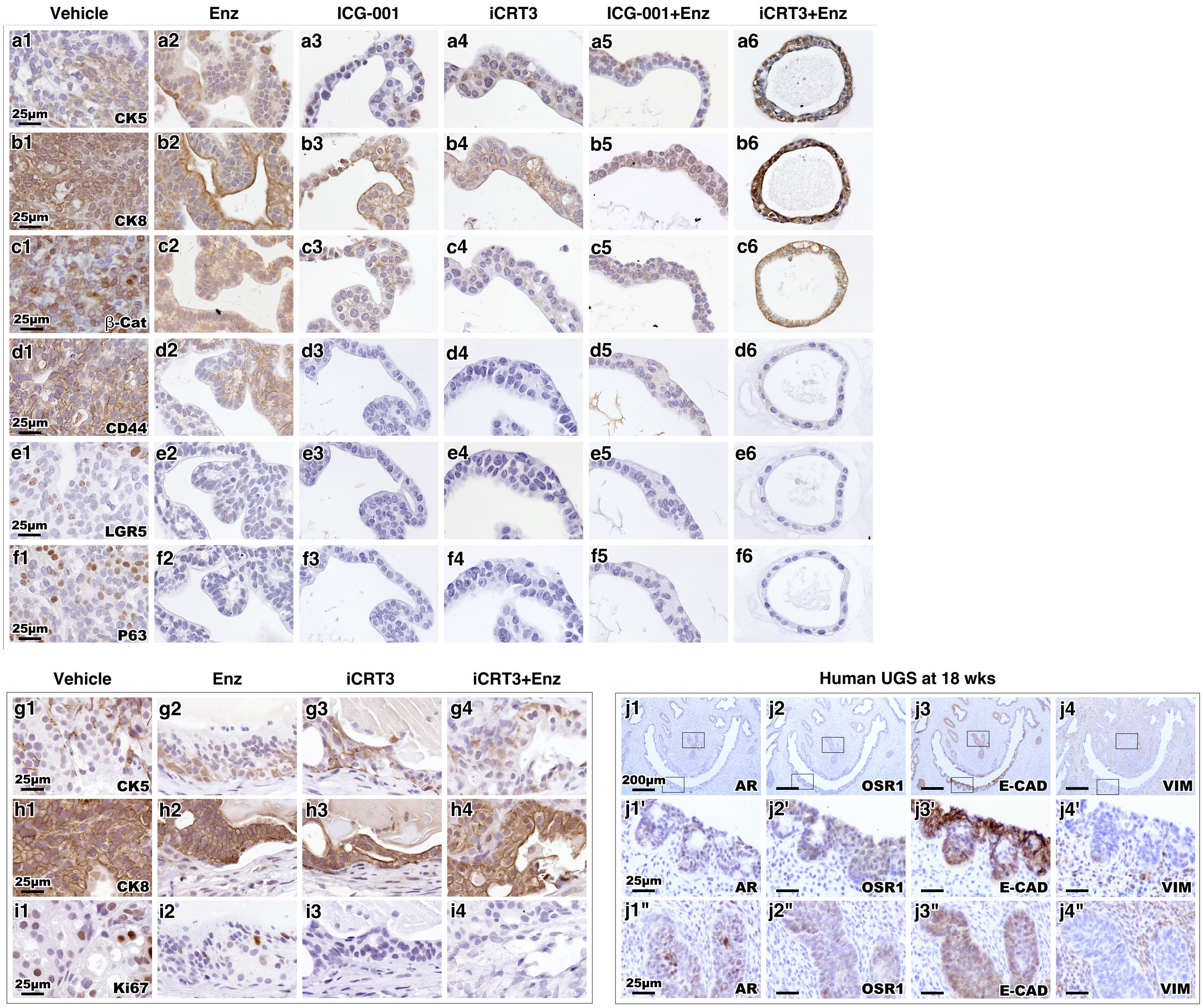

Supplementary Fig. 7 (Related to Fig. 8). Effect of AR or Wnt inhibitors on growth of hAR-expressing PCa and cellular properties of Osr1 expressing cells in human UGS. a-f Representative images of IHC staining for the indicated antibodies in adjacent sections from organoids derived from *R26*$^{mTmG/hAR}$:*Osr1*$^{Cre/+}$ mice with the indicated treatments. Scale bars, 25 μm. **g-i** Representative images of IHC staining for the indicated antibodies in xenograft tissue sections derived from *R26*$^{mTmG/hAR}$:*Osr1*$^{Cre/+}$ mice with the indicated treatments. Scale bars, 25 μm. **j** Representative images of IHC staining for the indicated antibodies in adjacent human UGS tissue sections (18 weeks old). Scale bars, 200 μm (**j1-4**); 25 μm (**j1'-4''**). Representative images with consistent results from three independent experimetns are shown.

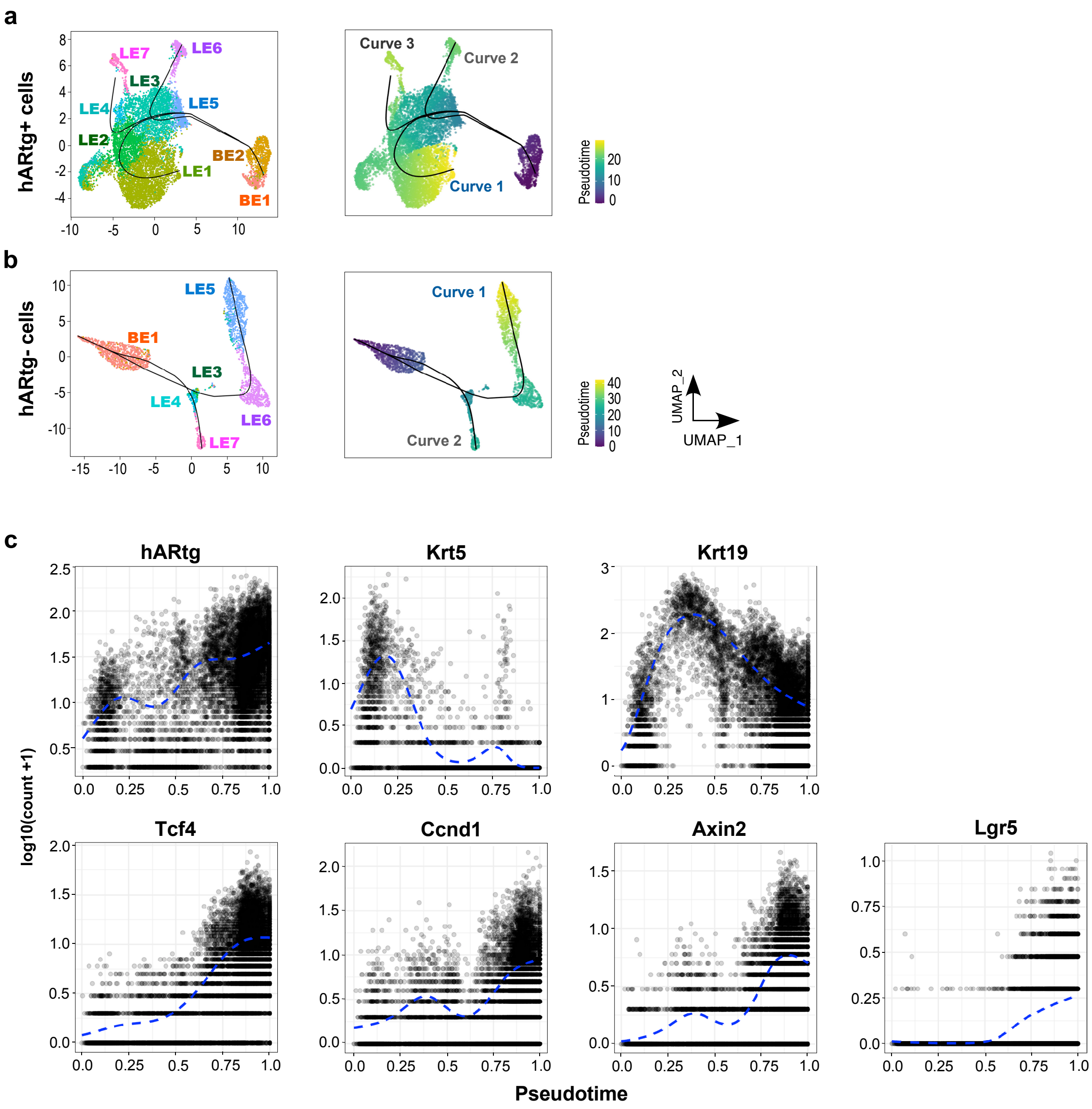

Supplementary Figure 8 (Related to Fig 7). Pseudotime Trajectory analysis using hARtg+ or hARtg- epithelial cells from prostatic PIN and tumor tissues. a-b Visualization of *hARtg*+ (**a**) or *hARtg*- (**b**) epithelial cells, colored by cluster identity (left) or by inferred pseudotime (right) using Slingshot, on a 2-dimensional UMAP embedding. The black curve denotes the inferred lineage. **c** Visualization of the trajectories of cellular markers, including *hARtg*, *Krt14*, and *Krt8*, and Wnt downstream target genes, including *Tcf4*, *Ccnd1*, *Axin2,* and *Lgr5*, respectively, in *hARtg*+ epithelial cells, using PseudotimeDE with negative binomial-generalized additive model (NB-GAM). Dashed blue lines are the fitted curves by NB-GAM.

Supplementary Table 1. **Pathological Abnormalities in the Prostates of Mice with Indicated Genotype**

Genotypes	< 6 M	6-12 M	>12 M
$R26^{mTmG/+}$:$Osr1^{Cre/+}$	12 of 12 Normal	10 of 10 Normal	8 of 8 Normal
$R26^{mTmG/hAR}$:$Osr1^{Cre/+}$	3 of 8 HGPIN	2 of 15 Intraductal adenocarcinomas<br>9 of 15 HGPIN	2 of 16 Invasive adenocarcinomas<br>5 of 16 Intraductal adenocarcinomas<br>10 of 16 HGPIN
$R26^{hAR/+}$:$Osr1^{Cre/+}$	5 of 13 HGPIN	3 of 18 Intraductal adenocarcinomas<br>10 of 18 HGPIN	2 of 17 Invasive adenocarcinoma<br>5 of 17 Intraductal adenocarcinoma<br>10 of 17 HGPIN

Supplementary Table 2. Information of Oligonucleotide Primers Used in This Study.

	Gene	Primer	Sequences
Genotyping	*R26*^{hAR}	Forward	5’ – AGC GCA TCG CCT TCT ATC GCC TTC - 3’
		Reverse	5’ – CTC TGG AAC AGA TTC TGG AAA GCT C - 3’
	R26^{mTmG}	Forward	5’ - CTC TGC TGC CTC CTG GCT TCT - 3’
		Reverse 1	5’ - TCA ATG GGC GGG GGT CGT T - 3’
		Reverse 2	5’ - CGA GGC GGA TCA CAA GCA ATA - 3’
	Osr1^{Cre+}	Forward	5’- GCT CCA GGT TTG TCT GAA TGT GG - 3’
		Reverse	5’- TCA TCA CTC GTT GCA TCG ACC - 3’
qPCR	*Ppia*	Forward	5’- TGT GCC AGG GTG GTG ACT TT - 3’
		Reverse	5’- CGT TTG TGT TTG GTC CAG CAT - 3’
	Tcf4	Forward	5’ - ATT TGT GGC CAT TGA AGG TT- 3’
		Reverse	5’ - GTC CCT AAG GCA GCC ATT C - 3’
	cMyc	Forward	5’ - CCC TAT TTC ATC TGC GAC GAG - 3’
		Reverse	5’ - TGG GAA GCA GCT CGA ATT T- 3’
	Ccnd1	Forward	5’ - GCG TAC CCT GAC ACC AAT CTC - 3’
		Reverse	5’ - ACT TGA AGT AAG ATA CGG AGG GC - 3’
	Axin2	Forward	5’ - ATG AGT AGC GCC GTG TTA GTG - 3’
		Reverse	5’ - GCA TAG GTT TGG TGG ACT GGA - 3’
	Lgr5	Forward	5’ - TGC CCA TCA CAC TGT CAC TGT - 3’
		Reverse	5’ - CAC CCT GAG CAG CAT CCT G - 3’
	Igf1r	Forward	5’ - TGA CAT CCG CAA CGA CTA TCA - 3’
		Reverse	5’ - CCA GTG CGT AGT TGT AGA AGA GT - 3’
	Jak2	Forward	5’ - TTG TGG TAT TAC GCC TGT GTA - 3’
		Reverse	5’ - ATG CCT GGT TGA CTC GTC TAT - 3’
	Mapk13	Forward	5’ - ATG AGC CTC ACT CGG AAA AGG - 3’
		Reverse	5’ - GCA TGT GCT TCA AGA GCA GAA - 3’
ChIP-qPCR	*Untr4*	Forward	5’ - CTC CCT CCT GTG CTT CTC AG - 3’
		Reverse	5’ - AAT GAA CGT GTC TCC CAG AA - 3’
	Igf1r	Forward	5’ - GAG GCT GAG GTT CTT GTT TAC CA- 3’
	binding site A	Reverse	5’ - CCT CTC TCG AGT TCG CCC G - 3’
	Igf1r	Forward	5’ - TCC CTT TGC TCC TAA ACA AGA TGA - 3’
	binding site B	Reverse	5’ - GGA CAC AAC AGC CTT AGA TGG - 3’

Supplementary Table 3. Information for Antibodies Used in This Study.

Antibody	Vendors and Cat #	Species	Working dilution
GFP	Cell Signaling #2956	rabbit IgG	1:200
GFP	Cell Signaling #2955	mouse IgG	1:200
GFP	Abcam #ab13970	chicken IgG	1:2000
OSR1	Biorbyt #orb162212	rabbit IgG	1:300
AR	ThermoFisher #PA1-9005	goat IgG	1:500
CK8	Convance #MMS-162P	mouse IgG	1:2000
CK8	Abcam #ab59400	Rabbit IgG	1:1000
CK5	Convance #PRB-160P	rabbit IgG	1:2400
P63	Biolegend #687202	mouse IgG	1:500
Vimentin	BioLegend #919101	chicken IgG	1:2000
SMA	Sigma Aldrich # A5228	mouse IgG	1:2000
hAR	Santa Cruz #sc-7305	mouse IgG	1:100
CK14	Abcam #ab7800	mouse IgG	1:200
IGF1R	Cell Signaling #9449	rabbit IgG	1:750
pIGF1R	Bioss Antibodies #bs-5447R	rabbit IgG	1:350
β-catenin	BD Transduction Laboratories #610154	mouse IgG	1:200
β-catenin	Santa Cruz #sc-7199	rabbit IgG	1:500
MYC	Abcam #ab168727	rabbit IgG	1:1000
TCF4	NovusBio #NBP2-67618	rabbit IgG	1:250
Cyclin D1	Abcam #ab16663	rabbit IgG	1:200
AXIN2	Abcam #ab32197	rabbit IgG	1:2000
LGR5	Abcam #ab219107	rabbit IgG	1:500
pAKT	Cell Signaling #9271	rabbit IgG	1:50
pGSK3	Cell Signaling #9331	rabbit IgG	1:200
p-ERK1/2	Cell Signaling #4370	rabbit IgG	1:100
Biotinylated anti-mouse	Vector Laboratories #BA-9200	goat IgG	1:750
Biotinylated anti-rabbit	Vector Laboratories #BA-1000	goat IgG	1:750
Biotinylated anti-goat	Vector Laboratories #BA-5000	rabbit IgG	1:750
Biotinylated anti-rat	Vector Laboratories #BA-9400	goat IgG	1:750
Goat anti-rabbit 488	Invitrogen #A11034	goat IgG	1:500
Goat anti-mouse 488	Invitrogen #A11001	goat IgG	1:500
Goat anti-rabbit 594	Invitrogen #A11012	goat IgG	1:500
Goat anti-mouse 594	Invitrogen #A11005	goat IgG	1:500
Goat anti-chicken 647	Invitrogen #A31571	goat IgG	1:500
Donkey anti-rabbit 488	Invitrogen #A21206	donkey IgG	1:500
Donkey anti-mouse 488	Invitrogen #A21202	donkey IgG	1:500
Donkey anti-rabbit 594	Invitrogen #A21207	donkey IgG	1:500
Donkey anti-mouse 594	Invitrogen #A21203	donkey IgG	1:500
Donkey anti-goat 647	Invitorgen #A21447	donkey IgG	1:500
hAR for ChIP-seq	Santa Cruz #sc-7305X	mouse IgG	1:100
