# Supplementary material for: Aberrant androgen action in prostatic progenitor cells induces oncogenesis and tumor development through IGF1 and Wnt axes
Source: Nat Commun. 2022 Jul 28;13:4364. doi: 10.1038/s41467-022-32119-0 (PMC9334353; doi:10.1038/s41467-022-32119-0)
Supplement: Supplementary file 3 — Description of Additional Supplementary Files [file 41467_2022_32119_MOESM3_ESM.pdf]

Description of Additional Supplementary Files

File Name: Supplementary Data 1
Description: The list of differentially expressed genes between *hARtg* positive and negative basal epithelial cells, related to Figure 3

File Name: Supplementary Data 2
Description: Differential binding regions from ChIP-seq data, related to Figure 3

File Name: Supplementary Data 3
Description: The list of differentially expressed genes between tumor and normal luminal epithelial cells, related to Figure 4

File Name: Supplementary Data 4
Description: The list of differentially expressed genes between PIN and WT, related to Figure 5

File Name: Supplementary Data 5
Description: The list of differentially expressed genes between PCa and WT, related to Figure 5

File Name: Supplementary Data 6
Description: The list of differentially expressed genes between PCa and PIN, related to Figure 5
