# Supplementary material for: Aberrant androgen action in prostatic progenitor cells induces oncogenesis and tumor development through IGF1 and Wnt axes
Source: Nat Commun. 2022 Jul 28;13:4364. doi: 10.1038/s41467-022-32119-0 (PMC9334353; doi:10.1038/s41467-022-32119-0)
Supplement: Supplementary file 10 — Reporting Summary [file 41467_2022_32119_MOESM10_ESM.pdf]

nature portfolio

Corresponding author(s): Zijie Sun; NCOMMS-21-36701B

Last updated by author(s): Jun 11, 2022

Reporting Summary

Nature Portfolio wishes to improve the reproducibility of the work that we publish. This form provides structure for consistency and transparency in reporting. For further information on Nature Portfolio policies, see our Editorial Policies and the Editorial Policy Checklist.

Statistics

For all statistical analyses, confirm that the following items are present in the figure legend, table legend, main text, or Methods section.

n/a	Confirmed	
☐	☒	The exact sample size (*n*) for each experimental group/condition, given as a discrete number and unit of measurement
☐	☒	A statement on whether measurements were taken from distinct samples or whether the same sample was measured repeatedly
☐	☒	The statistical test(s) used AND whether they are one- or two-sided<br>*Only common tests should be described solely by name; describe more complex techniques in the Methods section.*
☒	☐	A description of all covariates tested
☐	☒	A description of any assumptions or corrections, such as tests of normality and adjustment for multiple comparisons
☐	☒	A full description of the statistical parameters including central tendency (e.g. means) or other basic estimates (e.g. regression coefficient) AND variation (e.g. standard deviation) or associated estimates of uncertainty (e.g. confidence intervals)
☐	☒	For null hypothesis testing, the test statistic (e.g. *F*, *t*, *r*) with confidence intervals, effect sizes, degrees of freedom and *P* value noted<br>*Give P values as exact values whenever suitable.*
☒	☐	For Bayesian analysis, information on the choice of priors and Markov chain Monte Carlo settings
☒	☐	For hierarchical and complex designs, identification of the appropriate level for tests and full reporting of outcomes
☐	☒	Estimates of effect sizes (e.g. Cohen's *d*, Pearson's *r*), indicating how they were calculated

Our web collection on statistics for biologists contains articles on many of the points above.

Software and code

Policy information about availability of computer code

Data collection	Novaseq 6000 (Illumina) and Cell Ranger pipeline 3.1.0 (10× Genomics) were used for single-cell sequencing data collection. Hiseq 2500 (Illumina) was used for ChIP-seq and RNA-seq data collection. Axio Lab A1 microscope, Canon EOS 1000D camera with AxioVision software (Carl Zeiss), ECLIPSE E800 epifluorescence microscope (Nikon), and QImaging RETGA EXi camera with QCapture software (QImaging) were used for image. Included in methods section.
Data analysis	The bioinformatics analyses were conducted using open-source software, including Seurat version 3.2.1, R version 3.6.3, Novoalign version 3.02.07, MACS version 2.0, IGV version 6.3, cutadapt version 1.9.1, HISAT2 version 2.1.0, HTSeq version 0.6.1, edgeR version 2.26.7, GSEA version 4.1.0, and IPA version 01-20-04. R scripts used to process sequencing data are available upon request.

For manuscripts utilizing custom algorithms or software that are central to the research but not yet described in published literature, software must be made available to editors and reviewers. We strongly encourage code deposition in a community repository (e.g. GitHub). See the Nature Portfolio guidelines for submitting code & software for further information.

Data

Policy information about availability of data

All manuscripts must include a data availability statement. This statement should provide the following information, where applicable:

- Accession codes, unique identifiers, or web links for publicly available datasets
- A description of any restrictions on data availability
- For clinical datasets or third party data, please ensure that the statement adheres to our policy

Raw data of RNA-seq, single cell RNA-seq, ChIP-seq have been deposited in Gene Expression Omnibus database under accession number GSE164971.

Field-specific reporting

Please select the one below that is the best fit for your research. If you are not sure, read the appropriate sections before making your selection.

☒ Life sciences ☐ Behavioural & social sciences ☐ Ecological, evolutionary & environmental sciences

For a reference copy of the document with all sections, see nature.com/documents/nr-reporting-summary-flat.pdf

Life sciences study design

All studies must disclose on these points even when the disclosure is negative.

Sample size	Sample size estimated in this study was based on number of available, statistical methods, and previous reported data with related mouse models.
Data exclusions	None of the were data excluded. All of generated male mice were included in the study due to the nature of the prostate cancer models.
Replication	All experiments were performed at least three independent times and/or with sufficient samples per group for statistical significance. All replication attempts were successful.
Randomization	All of experimental mice with different genotypes were included in this study. For in vivo kidney capsule implantation, approximately 1×10^5 prostate adenocarcinoma cells were transplanted under the renal capsule of 6 to 8-week-old male NOD/SCID mice. 4 weeks after implantation, mice were randomized into vehicle control and treatment groups and were administered with vehicle (10% DMSO in corn oil) or iCRT3 (HY-103705, MedChemExpress) at 30 mg/kg body weight.
Blinding	Blinding methods were used for assessing the pathological/immunohistological changes in prostate tumor developments for each genotype/ mouse model used in this study.

Reporting for specific materials, systems and methods

We require information from authors about some types of materials, experimental systems and methods used in many studies. Here, indicate whether each material, system or method listed is relevant to your study. If you are not sure if a list item applies to your research, read the appropriate section before selecting a response.

Materials & experimental systems

n/a	Involved in the study	
☐	☒	Antibodies
☒	☐	Eukaryotic cell lines
☒	☐	Palaeontology and archaeology
☐	☒	Animals and other organisms
☒	☐	Human research participants
☒	☐	Clinical data
☒	☐	Dual use research of concern

Methods

n/a	Involved in the study	
☐	☒	ChIP-seq
☒	☐	Flow cytometry
☒	☐	MRI-based neuroimaging

Antibodies

Antibodies used	Included in Supplementary Table 9 and referred in methods section. All antibodies for immunohistochemistry, immonfluorescence, and immunoprecipitation used were from commercial sources: GFP, Cell Signaling #2956, rabbit IgG, 1:200; GFP, Cell Signaling #2955, mouse IgG, 1:200; GFP, Abcam #ab13970, chicken IgG, 1:2000; OSR1, Biorbyt #orb162212, rabbit IgG, 1:300; AR, ThermoFisher #PA1-9005, goat IgG, 1:500; CK8, Convance #MMS-162P, mouse IgG, 1:2000; CK8, Abcam #ab59400, rabbit IgG, 1:1000; CK5, Convance #PRB-160P, rabbit IgG, 1:2400; P63, Biolegend #687202, mouse IgG, 1:500; Vimentin, BioLegend #919101, chicken IgG, 1:2000; SMA, Sigma Aldrich # A5228, mouse IgG, 1:2000; hAR, Santa Cruz #sc-7305, mouse IgG, 1:100; CK14, Abcam #ab7800, mouse IgG, 1:200; IGF1R, Cell Signaling #9750, rabbit IgG, 1:750; pIGF1R, Bioss Antibodies #bs-5447R, rabbit IgG, 1:350; β-catenin, BD Transduction Laboratories #610154, mouse IgG, 1:200; β-catenin, Santa Cruz #sc-7199, rabbit IgG, 1:500; MYC, Abcam #ab168727, rabbit IgG, 1:1000; TCF4, NovusBio #NBP2-67618, rabbit IgG, 1:250; Cyclin D1, Abcam #ab16663, rabbit IgG, 1:200; AXIN2, Abcam #ab32197, rabbit IgG, 1:2000; LGR5, Abcam #ab219107, rabbit IgG, 1:500; pAKT, Cell Signaling #9271, rabbit IgG, 1:50; pGSK3, Cell Signaling #9331, rabbit IgG, 1:200; p-ERK1/2, Cell Signaling #4370, rabbit IgG, 1:100; hAR for ChIP-seq, Santa Cruz #sc-7305X, mouse IgG, 1:100; Biotinylated anti-mouse, Vector Laboratories #BA-9200, goat IgG, 1:750; Biotinylated anti-rabbit, ector Laboratories #BA-1000, goat IgG, 1:750; Biotinylated anti-goat, Vector Laboratories #BA-5000, rabbit IgG, 1:750; Biotinylated anti-rat, Vector Laboratories #BA-9400, goat IgG, 1:750; Goat anti-rabbit 488, Invitrogen #A11034, goat IgG, 1:500; Goat anti-mouse 488, Invitrogen #A11001, goat IgG, 1:500; Goat anti-rabbit 594, Invitrogen #A11012, goat IgG, 1:500; Goat anti-mouse 594, Invitrogen #A11005, goat IgG, 1:500; Goat anti-chicken 647, Invitrogen #A31571, goat IgG, 1:500; Donkey anti-rabbit 488, Invitrogen #A21206, donkey IgG, 1:500; Donkey anti-mouse 488, Invitrogen #A21202, donkey IgG, 1:500; Donkey anti-rabbit 594, Invitrogen #A21207, donkey IgG, 1:500; Donkey anti-mouse 594, Invitrogen #A21203, donkey IgG, 1:500; Donkey anti-goat 647, Invitorgen #A21447, donkey IgG, 1:500.

Validation

All antibodies used were from commercial sources and vendor confirmed species reactivity and references listed on vendor website: https://www.cellsignal.com/products/primary-antibodies/gfp-d5-1-rabbit-mab/2956; https://www.cellsignal.com/products/primary-antibodies/gfp-4b10-mouse-mab/2955; https://www.abcam.com/gfp-antibody-ab13970.html; https://www.scbt.com/p/osr1-antibody-c-8; https://www.thermofisher.com/antibody/product/Androgen-Receptor-Antibody-Polyclonal/PA1-9005; https://www.biolegend.com/fr-lu/products/cytokeratin-8-monoclonal-antibody-purified-10929; https://www.abcam.com/cytokeratin-8-antibody-ab59400.html; https://www.biolegend.com/en-us/products/keratin-5-polyclonal-antibody-purified-10956?GroupID=GROUP26; https://www.biolegend.com/en-us/products/purified-anti-tp63-antibody-13251; https://www.biolegend.com/en-us/products/purified-anti-vimentin-antibody-11598; https://www.sigmaaldrich.com/US/en/product/sigma/a5228?gclid=CjwKCAjw7rWKBhAtEiwAJ3CWLIHTsM2Q2VT0LlXP1ILlOQB6tvfJkIWbDlymh8U_4q7uGkqggJ7FChoCvTcQAvD_BwE; https://www.scbt.com/p/ar-antibody-441?gclid=CjwKCAjw7rWKBhAtEiwAJ3CWLDlmAzo9ZPvilEYmELHyMGp9-k_HPMCfVddk5hkACMXLreWsCzK-whoCLSMQAvD_BwE; https://www.abcam.com/cytokeratin-14-antibody-ll002-ab7800.html; https://www.cellsignal.com/products/primary-antibodies/igf-i-receptor-b-d23h3-xp-rabbit-mab/9750?site-search-type=Products&N=4294956287&Ntt=igf1r&fromPage=plp; https://www.biossusa.com/products/bs-5447r; https://www.bdbiosciences.com/en-us/products/reagents/microscopy-imaging-reagents/immunofluorescence-reagents/purified-mouse-anti-catenin.610153; https://www.scbt.com/p/beta-catenin-antibody-e-5?gclid=CjwKCAjw7rWKBhAtEiwAJ3CWLPlpOPZWTyHajWjrleCcHfwnw3hVSjtPoWQsBKhV_OAoSxZMfxMK8BoCeNIQAvD_BwE; https://www.abcam.com/c-myc-antibody-y69-bsa-and-azide-free-ab168727.html; https://www.novusbio.com/products/tcf7l2-antibody-sc06-90_nbp2-67618; https://www.abcam.com/cyclin-d1-antibody-sp4-ab16663.html; https://www.abcam.com/axin-2-antibody-ab32197.html; https://www.abcam.com/lgr5-antibody-ab219107.html; https://www.cellsignal.com/products/primary-antibodies/phospho-akt-ser473-antibody/9271; https://www.cellsignal.com/products/primary-antibodies/phospho-gsk-3a-b-ser21-9-antibody/9331; https://www.cellsignal.com/products/primary-antibodies/phospho-p44-42-mapk-erk1-2-thr202-tyr204-d13-14-4e-xp-rabbit-mab/4370; https://www.scbt.com/p/ar-antibody-441; https://vectorlabs.com/biotinylated-goat-anti-mouse-igg-antibody.html; https://vectorlabs.com/biotinylated-goat-anti-rabbit-igg-antibody.html; https://vectorlabs.com/biotinylated-rabbit-anti-goat-igg-antibody.html; https://vectorlabs.com/biotinylated-goat-anti-rat-igg-antibody.html; https://www.thermofisher.com/antibody/product/Goat-anti-Rabbit-IgG-H-L-Highly-Cross-Adsorbed-Secondary-Antibody-Polyclonal/A-11034; https://www.thermofisher.com/antibody/product/Goat-anti-Mouse-IgG-H-L-Cross-Adsorbed-Secondary-Antibody-Polyclonal/A-11001; https://www.thermofisher.com/antibody/product/Goat-anti-Rabbit-IgG-H-L-Cross-Adsorbed-Secondary-Antibody-Polyclonal/A-11012; https://www.thermofisher.com/antibody/product/Goat-anti-Mouse-IgG-H-L-Cross-Adsorbed-Secondary-Antibody-Polyclonal/A-11005; https://www.thermofisher.com/antibody/product/Donkey-anti-Mouse-IgG-H-L-Highly-Cross-Adsorbed-Secondary-Antibody-Polyclonal/A-31571; https://www.thermofisher.com/antibody/product/Donkey-anti-Rabbit-IgG-H-L-Highly-Cross-Adsorbed-Secondary-Antibody-Polyclonal/A-21206; https://www.thermofisher.com/antibody/product/Donkey-anti-Mouse-IgG-H-L-Highly-Cross-Adsorbed-Secondary-Antibody-Polyclonal/A-21202; https://www.thermofisher.com/antibody/product/Donkey-anti-Rabbit-IgG-H-L-Highly-Cross-Adsorbed-Secondary-Antibody-Polyclonal/A-21207; https://www.thermofisher.com/antibody/product/Donkey-anti-Mouse-IgG-H-L-Highly-Cross-Adsorbed-Secondary-Antibody-Polyclonal/A-21203; https://www.thermofisher.com/antibody/product/Donkey-anti-Goat-IgG-H-L-Cross-Adsorbed-Secondary-Antibody-Polyclonal/A-21447. All antibodies were further validated by IHC, IF, and ChIP prior to experiments.

Animals and other organisms

Policy information about studies involving animals; ARRIVE guidelines recommended for reporting animal research

Laboratory animals

All information for laboratory animals used are included in the methods section. Rosa26mTmG/+ (R26mTmG/+) reporter, Osr1-Cre, and Rosa26hARLoxP/wt, also named R26hAR/+ mice were obtained and used in this study, respectively. The PBCre/+ mice were obtained from the NCI mouse repository (strain #: 01XF5) . R26mTmG/hAR, R26mTmG/+:Osr1Cre/+, or R26mTmG/+:PbCre/+ mice were first produced and then used to generate R26mTmG/+:Osr1Cre/+ and R26mTmG/hAR:Osr1Cre/+ mice. Experimental mice generated in this study were mixed from C57BL/6 and 129S1/SvImJ backgrounds. 8-week-old male NOD.CB17-Prkdc(SCID)/_(Jax_001303) mice were used for kidney capsule grafting experiments.

Wild animals

This study did not involve wild animals.

Field-collected samples

This study did not involve samples collected from the field.

Ethics oversight

All experimental procedures and care of animals in this study were carried out according to the Institutional Animal Care and Use Committee (IACUC) at Beckman Research Institute at City of Hope, and approved by the IACUC. Euthanasia was performed by CO2 inhalation followed by cervical dislocation.

Note that full information on the approval of the study protocol must also be provided in the manuscript.

ChIP-seq

Data deposition

☒ Confirm that both raw and final processed data have been deposited in a public database such as GEO.

☒ Confirm that you have deposited or provided access to graph files (e.g. BED files) for the called peaks.

Data access links
May remain private before publication.

GSE164968
https://www.ncbi.nlm.nih.gov/geo/query/acc.cgi?acc=GSE164968
secure token: cvcjogskfnaxbeb

Files in database submission

ChIP-seq files in database submission with accession GSE164968.

Raw files
37299_Input_S7_L999_R1_001.fastq

37301_ARQ9-hAR_S9_L999_R1_001.fastq

Processed files
GSM5023996: 37299_Input.bedGraph.gz
GSM5023997: 37301_ARQ9-hAR.bedGraph.gz

Supplementary Fig. S5: Genomic location and enrichment profiles of hAR ChIP-seq
Supplementary Table S3: Differential binding regions from ChIP-seq data

Genome browser session (e.g. UCSC)	no longer applicable

Methodology

Replicates	All data are collected from two biological replicates for each sample.
Sequencing depth	A total of >45 mio high quality paired-end reads of 41bp per sample was subjected to quality check using FastQC v0.11.4. After quality check, high quality reads (>99%) were aligned using Novoalign v3.02.07, yielding >56% of uniquely aligned reads in both ChIP and Input samples.
Antibodies	Antibodies against hAR (Santa Cruz #sc-7305X, mouse IgG) and IgG (Santa Cruz #sc-2025, normal mouse IgG) were used in this study.
Peak calling parameters	MACS v2.0 47 was used to call Peaks using the corresponding negative control from R26mTmG/+:Osr1Cre/+ and to generate bed graph files for visualization of peaks.
Data quality	Total number of peak called for each dataset with p<0.001 and at least 2 fold enrichment.
Software	he ChIPseq data collection and analyses were conducted using open-source software, including FastQC v0.11.4, Novoalign version 3.02.07, MACS version 2.0 and IGV version 6.3.
